# Overexpression of a Novel Thermostable and Chloride-Tolerant Laccase from *Thermus thermophilus* SG0.5JP17-16 in *Pichia pastoris* and Its Application in Synthetic Dye Decolorization

**DOI:** 10.1371/journal.pone.0119833

**Published:** 2015-03-19

**Authors:** Huiping Liu, Yu Cheng, Bing Du, Chaofan Tong, Shuli Liang, Shuangyan Han, Suiping Zheng, Ying Lin

**Affiliations:** 1 School of Bioscience and Bioengineering, South China University of Technology, Guangzhou, Guangdong 510006, China; 2 College of Food Science and Engineering, South China Agricultural University, Guangzhou, Guangdong 510642, China; College of Medicine, University of South Florida, UNITED STATES

## Abstract

Laccases have been used for the decolorization and detoxification of synthetic dyes due to their ability to oxidize a wide variety of dyes with water as the sole byproduct. A putative laccase gene (*LacTT*) from *Thermus thermophilus* SG0.5JP17-16 was screened using the genome mining approach, and it was highly expressed in *Pichia pastoris*, yielding a high laccase activity of 6130 U/L in a 10-L fermentor. The *LacTT* open reading frame encoded a protein of 466 amino acid residues with four putative Cu-binding regions. The optimal pH of the recombinant LacTT was 4.5, 6.0, 7.5 and 8.0 with 2,2'-azino-bis(3-ethylbenzothazoline-6-sulfonic acid) (ABTS), syringaldazine (SGZ), guaiacol, and 2,6-dimethoxyphenol (2,6-DMP) as the substrate, respectively. The optimal temperature of LacTT was 90°C with guaiacol as the substrate. LacTT was highly stable at pH 4.0–11.0 and thermostable at 40°C–90°C, confirming that it is a pH-stable and thermostable laccase. Furthermore, LacTT also exhibited high tolerance to halides such as NaCl, NaBr and NaF, and decolorized 100%, 94%, 94% and 73% of Congo Red, Reactive Black B and Reactive Black WNN, and Remazol Brilliant Blue R, respectively. Interestingly, addition of high concentration of NaCl increased the RBBR decolorization efficiency of LacTT. These results suggest that LacTT is a good candidate for industrial applications such as dyestuff processing and degradation of dyes in textile wastewaters.

## Introduction

Laccases (benzenediol: oxygen oxidoreductases, EC 1.10.3.2) belong to the family of multi-copper oxidases (MCOs) that usually contain four copper atoms and are classified as blue copper proteins [1]. These enzymes couple the four-electron reduction of molecular oxygen to water with the oxidation of a wide variety of phenolic and non-phenolic compounds, including synthetic dyes, polyaromatic hydrocarbons (PAHs) such as NAP, and pesticides such as DDT, which are recalcitrant to natural degradation and are highly toxic and carcinogenic [2–4]. As conventional bioremediation methods are costly with low efficiency [3,5,6], laccases could be good candidates to detoxify these compounds. These versatile biocatalysts have attracted increasing interest in basic and applied research [7], and novel and engineered laccases are being developed to obtain an outstanding candidate with superior performance for “green” biotechnological applications [6,8,9].

In the recent decades, it has been reported that a number of fungal and bacterial laccases could decolorize and degrade industrial dyes, such as azo and anthroquinone dyes, which are widely used in the textile, leather, plastic, cosmetic, and food process industries [3,10]. The wastewater containing dye pollutants from textile industries usually has neutral or alkaline pH and contains high concentrations of chlorides, detergents, sulfate, and metal ions [11]. Unfortunately, the majority of the laccases from fungi such as *Paraconiothyrium variabile* [12], *Pleurotus florida* [13], and *Polyporus brumalis* [14] have low thermostability, lose their activities under alkaline conditions, and are susceptible to high concentrations of chloride [10,15]. These disadvantages of fungal laccases limit their practical applications. However, bacterial laccases may be attractive alternatives, because many of them present high pH stability and are resistant to high temperature and chlorides [10,16,17]. These properties could benefit the applications these enzymes in the decolorization of synthetic dyes from textile industries. Exemplarily, one of the most thermostable laccases, *Tth*-laccase from *Thermus thermophilus* HB27, has been well-studied and crystallized [17–19]. This enzyme has been reported to show extreme thermostability at 80°C with a half-life of more than 14 h [17], and has been applied in biobleaching of wheat straw pulp [20,23]. Other bacterial laccases from *Bacillus halodurans* [20], *Bacillus pumilus* [21], and *Streptomyces sviceus* [22] have also been found to exhibit thermostability or stability under alkaline conditions.

As the production of laccases is limited by low yield [6], it is important to search for potent laccases with efficient expression hosts. *Pichia pastoris*, which is now an established industrial platform for the production of heterologous proteins, is one of the most effective and ideal expression systems for achieving high yield of extracellular proteins [23,24]. To improve the yield of the expressed proteins in *P*. *pastoris*, generation of multicopy expression cassette strains is one of the most efficient ways [25].

In the present study, we obtained a putative laccase gene from *T*. *thermophilus* SG0.5JP17–16 by genome mining, which differed by 25% in its amino acid sequence from *T*. *thermophilus* HB27 laccase. We cloned and overexpressed the putative laccase gene in *P*. *pastoris*, and the recombinant protein was subsequently purified and biochemically characterized. The laccase from *T*. *thermophilus* SG0.5JP17–16 exhibited outstanding ability to decolorize synthetic dyes.

## Materials and Methods

### Strains, media and chemicals

*Escherichia coli* Top10 (Invitrogen, Carlsbad, CA, USA) was used for plasmid amplification and *P*. *pastoris* GS115 (Invitrogen, Carlsbad, CA, USA) was employed for the heterologous expression of the recombinant laccase. The *P*. *pastoris* expression vectors pHKFA were constructed in our laboratory. *E*. *coli* was grown in Luria-Bertani (LB) medium at 37°C and 200 rpm. *P*. *pastoris* was grown in BMGY/BMMY medium (1% yeast extract, 2% peptone, 100 mM potassium phosphate, 1.34% yeast nitrogen base (YNB), and 1% glycerol or 1% methanol; pH 6.0) at 30°C and 250 rpm. Complemented integrants (His^+^) were selected on MD solid medium (1.34% YNB, 4×10^-5^% (w/v) biotin, and 2% (w/v) glucose). *Pichia* trace metal (PTM_1_) salts (CuSO_4_·5H_2_O 0.6%, KI 0.009%, MnSO_4_·H_2_O 0.3%, H_3_BO_3_ 0.002%, MoNa_2_ O_4_·2H_2_O 0.024%, CoCl_2_ 0.05%, ZnCl_2_ 2%, FeSO_4_·H_2_O 6.5%, biotin 0.02%, and H_2_SO_4_ 0.5%) and basal salt medium (BSM) (glycerol 4%, K_2_SO_4_ 1.82%, MgSO_4_·7H_2_O 1.49%, KOH 0.413%, H_3_ PO_4_ 2.67%, and CaSO_4_ 0.093%, PTM_1_ 0.435%) were used in fed-batch cultivation. NH_4_OH (28% v/v) was used as a nitrogen source and for pH adjustment. A 50% (w/v) glycerol solution containing 1.2% (v/v) PTM_1_, and 100% methanol solution containing 1.2% (v/v) PTM_1_ were used as feed solutions according to the protocol of ‘Pichia fermentation process guideline’ (Invitrogen, San Diego, CA, USA).

The chemicals 2,2'-azino-bis(3-ethylbenzothazoline-6-sulfonic acid) (ABTS), guaiacol, syringaldazine (SGZ), and 2,6-dimethoxyphenol (2,6-DMP) were purchased from Sigma—Aldrich (St. Louis, MO, USA). KOD FX DNA polymerase was purchased from Toyobo (Japan). T4 DNA ligase and restriction enzymes were purchased from TaKaRa (Dalian, China) or Fermentas (St. Leon-Rot, Germany), and QIA quick Gel Purification Kit was purchased from Qiagen (Qiagen Co., Hilden, Germany). All of the other chemicals used were of analytical grade.

### Vector construction and yeast transformation

A protein BLAST search (http://blast.ncbi.nlm.nih.gov/Blast.cgi) for uncharacterized bacterial laccases was conducted using a thermostable laccase (YP_005339) from *T*. *thermophilus* HB27 as the search template. Out of the numerous homologs, a protein (YP_005641270) annotated as “multicopper oxidase type 3” from *T*. *thermophilus* SG0.5JP17–16 was selected. SignalP 4.1 (http://www.cbs.dtu.dk/services/SignalP/) was employed to identify the signal peptide. The molecular weight and pI value were predicted by Expasy Proteomics server (www.expasy.org).

The sequence of the selected gene was optimized for *P*. *pastoris* expression using OptimumGene technology and synthesized by GenScript Biotech Co. (Nanjing, China). Amplification of the putative laccase gene was performed by PCR using the primers P_TTF_/P_TTR_ (Table 1) and the synthesized laccase gene as the template. The PCR product was purified by using the Gel Extraction Kit, digested with *EcoR*I and *Kpn*I, and then ligated into *EcoR*I/*Kpn*I-digested pHKFA vector. The ligation mixture was then transformed into *E*. *coli* TOP10 competent cells.

**Table 1:** Primers used for plasmid construction and qPCR analysis.

Name	Sequence(5’-3’)	Annotation
P_TTF_	GGAATTCAATACCGATAGAAGAACCCT	*Eco*RI site (underlined)
P_TTR_	GGGGTACCTTAGTGGTGGTGGTGGTGGTGGTGGTGTCCGACTTCCAAAACTCC	*Kpn*I site(underlined)
Pg1	GAATGCGGCCGCGGCAGCGTGCAGC	*Not*I site (underlined)
Pg2	GCTGGCGGCCGCCCTAGGGAATTCC	*Not*I site (underlined)
Prtl1	ACGGTAAAGAGGGAAATT	qPCR for *LacTT*
Prtl2	AAGCGATAAGGTACAAAGGA	qPCR for *LacTT*
Prtg1	GTCGGGACACGCCTGAAACT	qPCR for G fragment
Prtg2	CCACCTTTTGGACCCTATTGAC	qPCR for G fragment

The digestion of pHKFA-*LacTT* with *Bgl*II and *Bam*HI released the expression cassette (*AOX1* promoter plus *LacTT* gene). The *Bgl*II–*Bam*HI DNA fragment from pHKFA-*LacTT* was subcloned into the *Bam*HI site of pHKFA-*LacTT* to generate the two-copy plasmid pHKFA-(*LacTT*)_2_. This process was repeated to generate the four-copy plasmid pHKFA-(*LacTT*)_4_. The desired recombinant plasmid pHKFA-*LacTT* and pHKFA-(*LacTT*)_4_ were linearized using *Kpn2*I, purified, and transformed into *P*. *pastoris* GS115 competent cells by electroporation (Bio-Rad, USA) at 1.5 kV with a 0.2-cm cuvette. Positive clones were selected on an MD plate and incubated at 30°C. The integration of the laccase gene *LacTT* into the genome of *P*. *pastoris* was confirmed by colony PCR using the primers 5′-AOX1/3′-AOX1 (data not shown).

### Determination of the *LacTT* copy number by quantitative PCR

The quantitative PCR (qPCR) assay protocol was derived from the Pfaffl method [26]. A standard plasmid, pHKFA-G-*LacTT*, containing a 600-bp G fragment (the interval partial sequence between *P*. *pastoris* GS115 genes 8198905 and 8198906) and a *LacTT* gene was constructed. The G fragment was amplified from *P*. *pastoris* GS115 genomic DNA by PCR, using the primers Pg1/Pg2 (Table 1), and then cloned into pHKFA-*LacTT* to generate the standard plasmid pHKFA-G-*LacTT*. To prepare the standard plasmid working solution for qPCR assay, pHKFA-G-*LacTT* was diluted with ultrapure water to a 1 ng/μl solution. The qPCR assay was performed using a gradient dilution of pHKFA-G-*LacTT* working solution (from 1×10^-1^ to 1×10^-7^ ng/μl) as template and the primers Prtl1/Prtl2 and Prtg1/Prtg2 (Table 1). For each gradient sample, the crossing points of the amplification curve with the threshold line (C_T_) vs. the pHKFA-G-*LacTT* concentration input were plotted to calculate the slope. The yeast recombinant DNA and standard plasmid were analyzed simultaneously using an Applied Biosystems 7500 fast real-time PCR instrument (Applied Biosystems Inc., Foster City, CA, USA). For analyzing each gene of different strains, the data were subjected to the method developed by Sun et al. [27].

### Heterologous expression of LacTT in *P*. *pastoris*

Both shake flask cultivation and 10-L fed-batch fermentation were conducted to express the recombinant LacTT. For shake flask cultivation, single colonies with the laccase gene *LacTT* were inoculated and grown in 50-mL flasks containing 10 mL of BMGY medium at 30°C and 250 rpm. When the OD_600_ of the culture reached about 6.0, the cells were harvested by centrifugation at 5000×g and 4°C for 5 min and then resuspended in 50 mL of BMMY medium in 500-mL flasks containing 0.1 mM CuSO_4_ at an initial OD_600_ of 1.0. The culture was cultivated at 30°C and 250 rpm for 7 days for extracellular laccase production with daily addition of 1% methanol.

Alternatively, a 10-L standard mechanically agitated fermentor was employed for large-scale production of the enzyme with an initial medium volume of 5 L (FUS10-A, Shanghai Guoqiang Bioengineering Equipment Co., Ltd., Shanghai, China). Typical recombinant *P*. *pastoris* fermentation comprised three phases. The entire cultivation started with a batch phase (phase I) in BSM for initial cell growth, which lasted for about 18–24 h at 30°C and pH 5.5. After the glycerol in the medium was exhausted, the fed-batch phase (phase II) was initiated by feeding limited glycerol to allow further cell growth. When OD_600_ reached approximately 300, the pH of the broth was adjusted to 6.0 by adding ammonia solution (25%, v/v). The induction phase (phase III) was started by the addition of 10–15 g/h mixtures of glycerol and methanol (100% methanol: 50% glycerol = 1:4, v/v) as carbon source. The mixture feed rate was then adjusted upwards every 1 h until at 30 ± 5 g/h finally while DO kept constantly at about 20–30%. The fermentation was terminated after induction for 144 h, and the culture was centrifugation at 5000 ×g and 4°C for 5 min. The supernatant was collected for further experiments.

### Purification and characterization of the recombinant LacTT

The recombinant laccase was purified by immobilized metal affinity chromatography (IMAC), and the purification was performed with HisTrap FF crude column (GE Healthcare) using AKTA purifier FPLC system (GE Healthcare). Imidazole and sodium chloride were removed using HiTrap desalting columns (GE Healthcare) according to the manufacturer’s instructions. Elution was conducted with 50 mM Na_2_HPO_4_–NaH_2_PO_4_ buffer (pH 7.5) as the mobile phase. Purified LacTT was stored at 4°C. The purity of the purified enzyme was checked by sodium dodecyl sulfate polyacrylamide gel electrophoresis (SDS-PAGE), using 10% SDS-polyacrylamide gels. LacTT was heat-denatured by treating at 100°C for 5min in denaturing buffer (containing 1% SDS and 0.5% 2-Mercaptoethanol). The proteins were stained with Coomassie Brilliant Blue R-250. Zymogram analysis for laccase activity was performed using unheated protein on 10% SDS-polyacrylamide gel and denatured LacTT was used as a negative control. The gel was stained with 0.1 mM SGZ or 2 mM guaiacol. Deglycosylation of the purified laccase was performed using PNGase F (New England Biolabs, Ipswich, MA) according to the instructions. The purified protein concentration was estimated using Bradford assay with bovine serum albumin (BSA) (Sigma-Aldrich) as the standard. The amount of LacTT in supernatant expressed in *P*. *pastoris* was quantified using SDS-PAGE with equal volume of BSA of known concentration as an external reference protein and the concentration of the LacTT band was analyzed using Quantity One software (Bio-Rad, USA).

### Laccase activity assay

The LacTT activity was measured at 90°C using 2 mM guaiacol (ε_465_ = 12,100 M^-1^cm-^1^, dissolved in 95% ethanol with guaiacol stock concentration of 20 mM) as the substrate. The 1mL enzyme assay mixture contained appropriately diluted purified LacTT, 2 mM guaiacol, 10 μM CuSO_4_ and 50 mM Na_2_HPO_4_–NaH_2_PO_4_ buffer (pH 7.5). After incubation at 90°C for 5 min, the mixture was cooled in ice bath for 1 min to stop the reaction and the absorbance was measured at 465 nm. LacTT was inactive by adding 1mM EDTA in the enzyme assay mixture and used as a negative control for all the assays or measurements. Alternative substrates for the measurement of laccase activity were: 1 mM ABTS (ε_420_ = 36,000 M^-1^ cm^-1^), 100 μM SGZ (ε_525_ = 65,000 M^-1^ cm^-1^, dissolved in dimethyl sulfoxide, DMSO with SGZ stock concentration of 1 mM) and 2 mM 2, 6-DMP (ε_468_ = 49,600 M^-1^ cm^-1^, dissolved in 50% ethanol with 2, 6-DMP stock concentration of 20 mM) at their pH optima, respectively [16,35]. One unit of enzyme was defined as the amount of enzyme that oxidizes 1 μmol of substrate per minute. All of the assays were performed in triplicate.

### Characterization of the recombinant LacTT

The effects of pH on LacTT activity towards ABTS, SGZ, guaiacol, and 2,6-DMP were evaluated at 90°C over a pH range of 3.0–10.0 using Britton—Robinson buffer. The pH stability of LacTT was analyzed by measuring the residual enzyme activities after pre-incubation at 30°C for 12 h in Britton—Robinson buffer at different pH.

To measure the effect of temperature on enzyme activity, LacTT was incubated at a temperature range of 40°C–100°C using guaiacol as the substrate at pH 7.5. The thermostability of the enzyme was determined by incubating LacTT at various temperatures (40°C–90°C) for 1 h and at 70°C, 80°C, and 90°C for 4 h. The residual laccase activity was determined using guaiacol as the substrate.

The kinetic parameters for LacTT were measured at 90°C using different concentrations of ABTS (31.25–500 μM), SGZ (3.125–200 μM), guaiacol (62.5–4000 μM), and 2,6-DMP (62.5–4000 μM). The experimental results were fitted to the Lineweaver—Burk plots.

The effects of 1 mM K^+^, Li^+^, Mn^2+^, Mg^2+^, Co^2+^, Zn^2+^, Ca^2+^, Pb^2+^, SDS, and 0.1 mM or 1mM EDTA on the enzyme activity were investigated by incubating LacTT with each effector at pH 7.5 and room temperature for 15 min prior to the addition of guaiacol. A control without effector was also employed. The enzyme activity assay was performed similar to that described earlier with guaiacol.

### Effects of halides on LacTT activity

The effects of halides on LacTT activity were determined by adding NaCl, KCl, and NaBr at concentrations from 0 to 2000 mM and NaF at concentrations from 0 to 800 mM to the enzyme assay mixture with guaiacol as the substrate.

### Decolorization of synthetic dyes

Three azo dyes, namely, Reactive Black B (RBB, λ_max_ = 595 nm), Reactive Black WNN (RBWNN, λ_max_ = 594 nm), and Congo Red (CR, λ_max_ = 488 nm), and one anthraquinone dye Remazol Brilliant Blue R (RBBR, λ_max_ = 594 nm) were selected to evaluate the ability of LacTT to decolorize industrial synthetic dyes at 70°C. The decolorization reaction mixtures (2 mL) contained 50 mM Na_2_HPO_4_–NaH_2_PO_4_ buffer (pH 7.5, but pH 8.0 for CR decolorization), 50 mg/L dye, 10 μM CuSO_4,_ and purified laccase (40 U/L). The effects of NaCl on RBBR decolorization by LacTT were determined using 0–1 M NaCl. The reactions were conducted at 70°C for 24 h. The samples were analyzed spectrophotometrically at the maximal absorbance wavelength of each dye. All of the reactions were performed in triplicate.

## Results and Discussion

### Cloning and overexpression of *T*. *thermophilus* SG0.5JP17–16 laccase in *P*. *pastoris*

The novel laccase of *T*. *thermophilus* SG0.5JP17–16 was identified by Protein Blast using the laccase sequence of *T*. *thermophilus* HB27 as the search template. The uncharacterized bacterial laccase gene was designated as *LacTT*. The open reading frame of *LacTT* consisted of 1398 nucleotides encoding a protein of 466 amino acids. The theoretical molecular weight and pI of LacTT were 53 kDa and 8.82, respectively. LacTT exhibited only 75% amino acid identity to the laccase from *T*. *thermophilus* HB27. Multiple sequence alignment of LacTT with other fungal and bacterial laccase sequences demonstrated four conserved histidine-rich copper-binding domains in LacTT.

**Fig 1:**
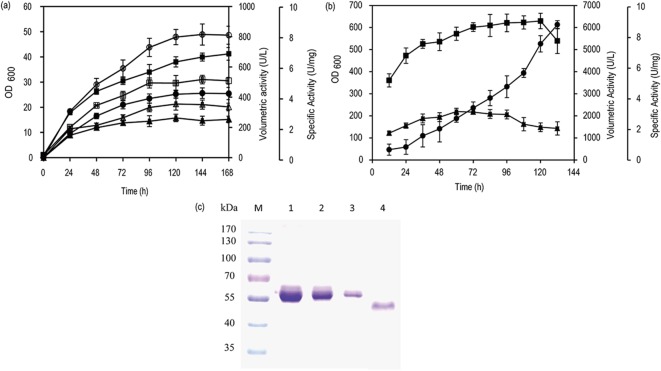
Time course of laccase production during shake flask cultivation and 10-L fed-batch fermentation. (a) Progress curves constructed in the induction phase of shake flask cultivation for the determination of the cell density of *P*. *pastoris/LacTT* (closed square) and *P*. *pastoris/*(*LacTT*)_4_ (open square), volumetric activity of *P*. *pastoris/LacTT* (closed circle) and *P*. *pastoris/*(*LacTT*)_4_ (open circle), specific activity of *P*. *pastoris/LacTT* (closed triangle) and *P*. *pastoris/*(*LacTT*)_4_ (open triangle). (b) Cell density (closed square), volumetric activity (closed circle), and specific activity (closed triangle) of *P*. *pastoris/*(*LacTT*)_4_ in fed-batch fermentation. (c) SDS-PAGE image of LacTT supernatant expressed in *P*. *pastoris/*(*LacTT*)_4_ in fed-batch fermentation. Lane M: protein marker; Lane 1: 0.5 mg/mL BSA; Lane 2: 0.3 mg/mL BSA; Lane 3: 0.1 mg/mL BSA; Lane 4: Enzyme supernatant was 1:10 diluted. The error bars represent the standard deviation.

The *LacTT* gene was cloned into a pHKFA vector under the control of alcohol oxidase 1 (*AOX1*) promoter. The recombinant *P*. *pastoris* cells bearing one or four copies of *LacTT* gene were obtained by transforming the recombinant plasmids pHKFA-*LacTT* and pHKFA-(*LacTT*)_4_ into *P*. *pastoris*. The *LacTT* copy numbers of the transformants were confirmed by qPCR assays, which indicated *P*. *pastoris*/*LacTT* with a copy of *LacTT* gene and *P*. *pastoris*/(*LacTT*)_4_ with four copies of *LacTT* (S1 Fig.). Both *P*. *pastoris/LacTT* and *P*. *pastoris/*(*LacTT*)_4_ were induced to produce laccase in shake flasks, and the laccase volumetric activity of *P*. *pastoris/*(*LacTT*)_4_ reached 813 U/L, which was about 1.9 fold higher than the maximum laccase volumetric activity of *P*. *pastoris/LacTT* (426 U/L) (Fig. 1a). The laccase-producing capacity of *P*. *pastoris/*(*LacTT*)_4_ was further evaluated by high-density fermentation in a 10-L fermentor. The maximal laccase volumetric activity of 6130 U/L of fed-batch fermentation was reached at 132h with a specific activity of 2.1 U/mg and LacTT yield of 1.2 g/L (Fig. 1b,c). To date, only a few bacterial laccases have been expressed in *P*. *pastoris*, and their laccase yield were low. For instance, the expression of a laccase from *B*. *licheniformis* in *P*. *pastoris* yielded only 227.9 U/L of laccase activity [10].

### Characterization of the purified laccase

**Fig 2:**
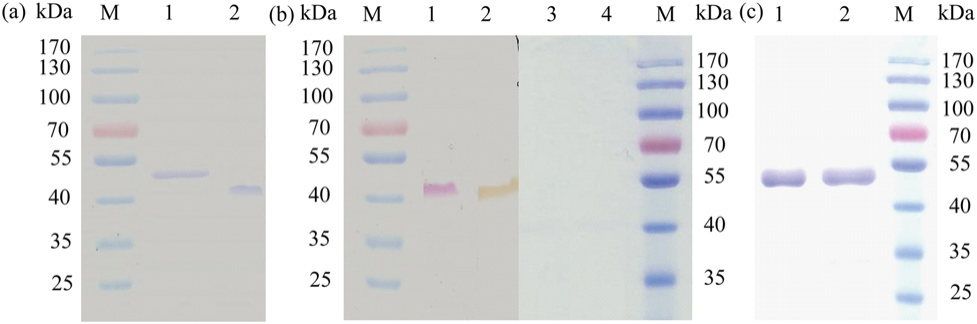
SDS-PAGE, zymogram analyses and glycosylation analyses of the purified LacTT. (a) The gel stained with Coomassie Brilliant Blue R-250. Lane M: protein marker; Lane 1: purified LacTT after heat denaturation; Lane 2: purified LacTT without heat denaturation. (b) The zymogram stained with 0.1 mM SGZ and 2 mM guaiacol. Lane M: protein marker; Lane 1: unheated purified LacTT stained with 0.1 mM SGZ; Lane 2: unheated purified LacTT stained with 2 mM guaiacol; Lane 3: heat-denatured LacTT stained with 0.1 mM SGZ; Lane 4: heat-denatured LacTT stained with 2 mM guaiacol. (c) Deglycosylation analyses of the purified laccase. Lane 1: purified LacTT; Lane 2: purified LacTT was deglycosylated by PNGase F; Lane M: protein marker.

The recombinant LacTT was purified by IMAC. SDS-PAGE analysis of the purified laccase showed that the recombinant LacTT was about 53 kDa, which was in agreement with its theoretical molecular mass without glycosylation (Fig. 2a, c). LacTT without heat denaturation was smller than the heat-denatured one in SDS-PAGE gel. We infer that the 53-kDa band of heat-denaturation represents the fully denatured protein and suggest that the faster migrating 46-kDa band without heat denaturation represents a correctly folding form of the enzyme, which remains its laccase activity and migrates faster (Fig. 2b). It is an interesting phenomenon that some laccases without heat denaturation may have a difference in mobility from the heat-denatured ones in SDS-PAGE gel [6, 10]. The zymogram analysis further confirmed the laccase identity of the purified protein, revealing that LacTT exhibited activity towards guaiacol and SGZ (Fig. 2b). The purified LacTT had a specific activity of 4.21, 1.12, 0.9, and 0.16 U/mg towards guaiacol, SGZ, ABTS, and 2,6-DMP, respectively, similar to the bacterial laccase Lac591 from uncultured bacteria, whose specific activity was 0.51, 0.15, 12.7, and 1 U/mg towards SGZ, ABTS, 2,6-DMP, and guaiacol, respectively [28]. However, the laccase CotA from *B*. *licheniformis* had been reported to exhibit a higher specific activity of 16 and 18.7 U/mg towards ABTS and SGZ, respectively [29].

**Fig 3:**
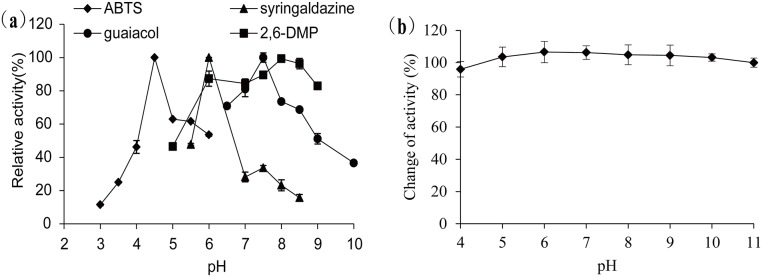
Effect of pH on the activity (a) and stability (b) of purified LacTT at 90°C. (a) ABTS, pH 3.0–6.0; SGZ, pH 5.5–8.5; guaiacol, pH 6.5–10.0; 2, 6-DMP, pH 5.0–10.0. (b) Investigation of the pH stability of LacTT by measuring the enzyme activity at 90°C with guaiacol as the substrate. The data represent the average values from triplicate measurements. The error bars represent the standard deviation.

The optimal pH for LacTT activity towards ABTS, SGZ, guaiacol, and 2,6-DMP was 4.5, 6.0, 7.5, and 8.0, respectively (Fig. 3a). This difference in the pH optimum for different substrates is typical for laccases. Furthermore, LacTT was quite stable over a pH range of 4.0–11.0, maintaining more than 95% of its original activity after incubating at 30°C for 12 h (Fig. 3b). While the majority of the bacterial laccases have been observed to exhibit an optimal activity towards SGZ at the pH range of 5.5–8.4, the optimum pH of the fungal laccases has been noted to fall within the range of 3.5–5.0 [2,30]. For bacterial laccases, the optimal pH for activity towards ABTS has been reported to be in the range of 4–6; however, most of the fungal laccases have been found to display a lower pH optimum (pH 2–5) [31].

**Fig 4:**
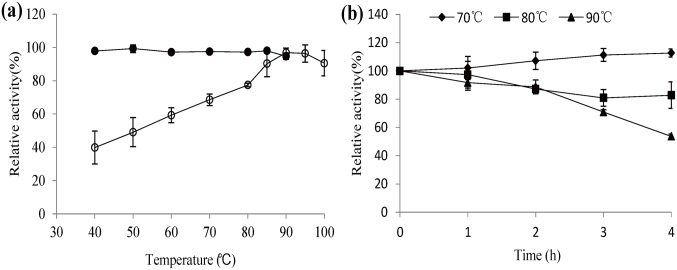
Effect of temperature on the activity and stability of the purified laccase. (a) Optimal temperature (open circles) was determined at pH 7.5 by using guaiacol as the substrate. Residual activity (closed circles) was determined after incubation at 40°C–90°C for 1 h. (b) Residual activity was determined after incubation at 70°C, 80°C, and 90°C, respectively, for 0–4 h. The graphs display the average values from triplicate measurements. The error bars represent the standard deviation.

The optimal reaction temperature of LacTT with guaiacol as the substrate was 90°C. At this temperature, more than 90% of the enzyme activity was maintained after 1 h of incubation (Fig. 4a). Furthermore, the LacTT activity increased to 113% of its original activity at 70°C and maintained more than 75% of its original activity at 80°C after 4 h of incubation (Fig. 4b). Thus, it can be concluded that LacTT is one of the most thermostable laccases reported so far, and its high thermo-alkali-stability indicates its potential application in industries.

The kinetic characteristic of LacTT for ABTS, SGZ, guaiacol, and 2,6-DMP oxidation was determined at its respective optimal pH at 90°C, and the results are shown in Table 2. The kinetic parameters and specific activity of LacTT for various substrates were noted to fall within the range reported in the literature for other bacterial laccases [28,32]. The highest catalytic efficiency of LacTT was found for SGZ, with lower *K*_*m*_ value and higher *k*_*cat*_/*K*_*m*_ value than those for the other substrates. Despite the high similarity between LacTT and *Tth*-laccase from *T*. *thermophilus* HB27, the catalytic features were different [17]. LacTT presented a much lower *K*_*m*_ value with SGZ and ABTS as the substrate, when compared with *Tth*-laccase. For SGZ oxidation by LacTT, the catalytic specificity (*k*_*cat*_/*K*_*m*_) was higher by a factor of 11 compared to *Tth*-laccase. Thus, the minor sequence variations in these two enzymes might play an important role in substrate binding and oxidation.

**Table 2:** Comparison of the kinetic properties of purified LacTT with those of laccase from *T*. *thermophilus* HB27.

Substrate	LacTT			*Tth*-laccase [17]		
	*K*_*m*_ (μM)	*k*_cat_ (s^-1^)	*k*_cat_/*K*_m_ (s^−1^μM^−1^)	*K*_*m*_ (μM)	*k*_cat_ (s^-1^)	*k*_cat_/*K*_m_ (s^−1^μM^−1^)
SGZ	27.0±1.5	1.001±0.061	0.037±0.0027	1880	6.47	0.0034
Guaiacol	363.3±29.1	4.345±0.216	0.012±0.001	n.a.	n.a.	n.a.
ABTS	36.0±3.4	0.365±0.012	0.011±0.001	900	24.6	0.027
2,6-DMP	152.1±13.8	0.13±0.012	0.001±0.00003	n.a.	n.a.	n.a.

n.a.: not available.

**Fig 5:**
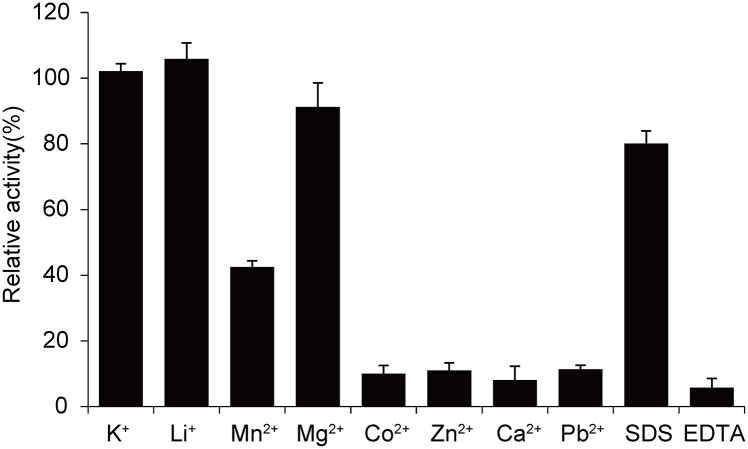
Effect of metal ions, SDS, and EDTA on LacTT activity. The laccase activity was determined at 90°C in 50 mM Na_2_HPO_4_-NaH_2_PO_4_ (pH 7.5, supplemented with 10 μM CuSO_4_) using guaiacol as the substrate. The error bars represent the standard deviation.

It has been reported that metal ions, EDTA, and detergents are inhibitors of laccases, because these agents are suggested to affect the laccase activity by chelating the Cu(II) atoms or modifying the amino acid residues [33]. In the present study, the effect of different metal ions, SDS, and EDTA on LacTT activity was also investigated (Fig. 5). The results revealed that in the presence of 1 mM K^+^ or Li^+^, the LacTT activity was stimulated up to 102% and 106%, respectively. The addition of 1mM Mg^2+^ caused slight decrease in LacTT activity to 91%. Whereas, the addition of 1 mM Mn^2+^ significantly reduced the LacTT activity to 42%, and Co^2+^, Zn^2+^, Ca^2+^, and Pb^2+^ dramatically reduced the LacTT activity to 10%, 11%, 8% and 11%, respectively. In addition, when 0.1 mM EDTA was applied the activity of LacTT was significantly reduced to 6% (Fig. 5). In the presence of 1 mM EDTA no laccase activity was observed, indicating that Cu^2+^ is essential for laccase activity. However, only 19% enzyme activity was lost when 1 mM SDS was added (Fig. 5).

### Effects of halides on LacTT activity

**Fig 6:**
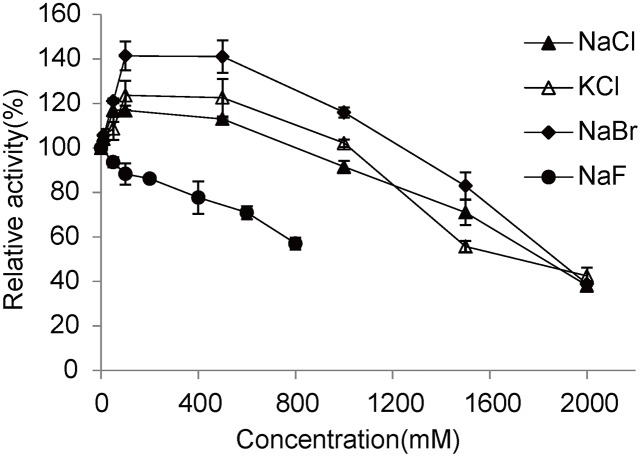
Effects of halides on LacTT activity. The enzyme was incubated in 50 mM Na_2_HPO_4_-NaH_2_PO_4_ (pH 7.5, supplemented with 10 μM CuSO_4_), using guaiacol as the substrate. The error bars represent the standard deviation.

Halides, especially chlorides are one of the major chemicals in textile wastewaters [34]. It has been suggested that halides and other anions such as azide, cyanide, and hydroxide bind to the T2/T3 coppers of laccases, resulting in an interruption of the internal electron transfer and subsequent inhibition of laccase activity [33]. Thus, these compounds may limit the application of laccases in decolorizing industrial dyes. In the present study, NaF, NaCl, and NaBr were used as the fluoride, chloride and bromide donators, respectively, to test the effects of halides on LacTT. Both NaCl and NaBr enhanced the activity of LacTT when their concentrations were less than 1 M. NaCl increased the activity of LacTT to 113% at concentrations from 50 to 500 mM, whereas NaBr increased the LacTT activity to 140% at concentrations of 100–500 mM. However, higher concentrations of NaCl and NaBr negatively influenced LacTT activity (Fig. 6). A similar effect was also exhibited by KCl (Fig. 6). LacTT lost half of its activity in the presence of about 1.9 M NaCl and NaBr. Furthermore, NaF decreased the enzyme activity at an I_50_ (the concentration of an inhibitor causing 50% activity reduction) of about 800 mM (Fig. 6). These findings clearly indicated that halides with smaller molecular radii were more efficient inhibitors of LacTT (fluoride > chloride> bromide).

It must be noted that LacTT represents one of the most halide-tolerant laccases reported so far. In previous studies, the I_50_ of NaCl towards the laccase from *Botrytis aclada* and laccase from an uncultured bacterium was noted to be 1.4 and 1.5 M, respectively, and the former enzyme lost 50% of its activity in the presence of 2.7 μM NaF [15,16]. In addition, the laccase from *Bacillus sp*. HR03 lost half of its activity in the presence of 0.7 M NaCl [35]. It has been reported that fungal laccases are more sensitive to NaCl. For instance, the laccase from *Trametes versicolor* lost 74% of its activity in the presence of 100 mM NaCl [36], while that from *Melanocarpus albomyces* was totally inhibited by the addition of 1 mM NaCl [37]. Thus, with the remarkable tolerance to halides, LacTT could be a valuable candidate for a wide range of industrial or environmental applications, such as dye decolorization of textile wastewaters in which the chloride concentration is always high [34].

### Decolorization of azo dyes and anthraquinone dye

**Fig 7:**
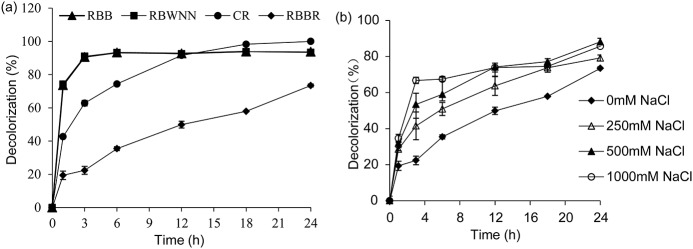
Decolorization of different dyes by LacTT. (a) Decolorization of synthetic dyes by LacTT at 70°C for 24 h. (b) Decolorization of RBBR by LacTT with different concentrations of NaCl at 70°C for 24 h. The reactions were performed in 50 mM Na_2_HPO_4_–NaH_2_PO_4_ buffer (pH 7.5, but pH 8.0 for CR decolorization), 50 mg/L dye, 10 μM CuSO_4,_ and purified LacTT (40 U/L). The error bars represent the standard deviation.

The azo and anthraquinone dyes are the major group of colorants used in the textile industry. In the present study, three azo dyes, namely, RBB, RBWNN, and CR, and one anthraquinone dye RBBR were selected to evaluate the ability of LacTT to decolorize synthetic dyes at 70°C. To date, there have been no reports on the use of laccases from *T*. *thermophilus* in synthetic dye decolorization. All of the four tested synthetic dyes were decolorized by the purified LacTT without the addition of expensive mediators. The decolorization of RBB and RBWNN readily reached 90% in the first 3 h, and the highest decolorization rate was finally reached (94%) after 24 h at 70°C (Fig. 7a). Furthermore, LacTT exhibited high efficiency in decolorizing CR at pH 8.0, with maximum decolorization activity observed after 24 h at 70°C (Fig. 7a), whereas about 73% of RBBR was decolorized after 24 h (Fig. 7a). In a previous study, it had been reported that about only 50% of RBB was decolorized by *B*. *licheniformis* laccase, with the decolorization rate reaching 92.79% with acetosyringone as the mediator [38]. In addition, the laccase (1.0 U mL^-1^) derived from *Trametes pubescens* was found to decolorize 80.53% of CR (50.0 mg L^-1^) after incubation for 72 h [39]. Thus, it can be concluded that LacTT has high decolorization efficiency.

The addition of NaCl obviously enhanced the RBBR decolorization efficiency of LacTT. More than 66% of RBBR decolorization was observed after the initial 3 h of incubation with the addition of 1 M NaCl, and the decolorization efficiency reached a maximum 88% after 24 h of incubation at 70°C with 500 mM NaCl (Fig. 7b). These results suggested that the decolorization activity of LacTT was stimulated rather than inhibited by NaCl up to a concentration of 1 M. As most of the textile effluents are characterized by high concentration of chlorides, a majority of the fungal laccases may lose their activities under this condition. It has been reported that both ABTS oxidation and dye decolorization of an anthraquinone dye (Reactive Blue 19) by the *T*. *versicolor* laccase were inhibited by NaCl. Therefore, the potential use of LacTT could be advantageous due to its high stability and decolorization efficiency under high NaCl concentration. The high efficiency of LacTT in decolorizing the examined synthetic dyes indicates that this enzyme might be a good candidate for purification of wastewaters from the textile industry.

## Conclusions

A novel bacterial laccase gene *LacTT* from *T*. *thermophilus* SG0.5JP17–16 was cloned and overexpressed in *P*. *pastoris*, which yielded a high laccase activity of 6130 U/L in a 10-L fermentor. The recombinant laccase LacTT was purified and characterized. The results showed that LacTT was tolerant to high temperatures, alkaline conditions, and halides. Moreover, LacTT displayed high efficiency in decolorizing synthetic dyes under alkaline conditions and even at high NaCl concentration, suggesting that this laccase is an environment-friendly candidate for use in the treatment of wastewaters from textile industry.

## Supporting Information

S1 Fig Quantitative PCR assay of the *LacTT* gene copy number in the genomic DNA of the recombinant yeast strains. The threshold value was set at 0.2. The values indicate the average ± standard deviations from triplicate qPCR experiments.
(TIF)
