# Supplementary figures and images for: Overexpression of a Novel Thermostable and Chloride-Tolerant Laccase from Thermus thermophilus SG0.5JP17-16 in Pichia pastoris and Its Application in Synthetic Dye Decolorization
Source: PLoS One. 2015 Mar 19;10(3):e0119833. doi: 10.1371/journal.pone.0119833 (PMC4366370; doi:10.1371/journal.pone.0119833)

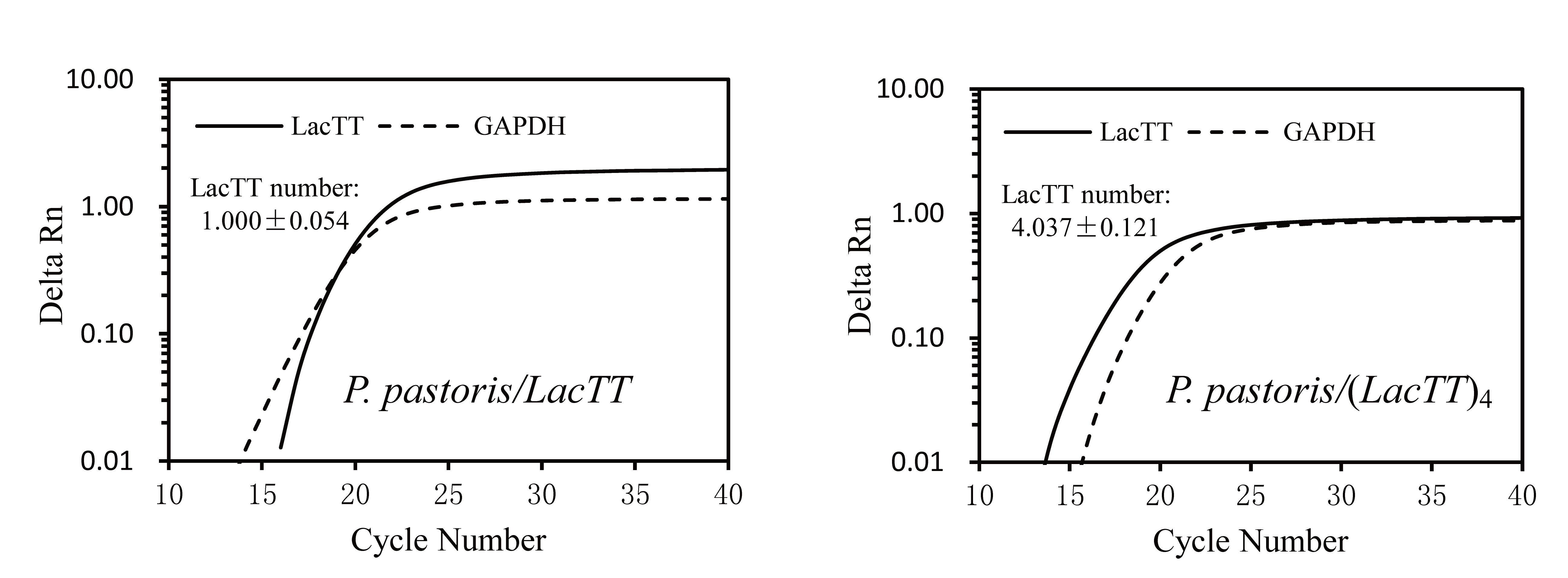

Supplement: S1 Fig — The threshold value was set at 0.2. The values indicate the average ± standard deviations from triplicate qPCR experiments. (TIF) [file pone.0119833.s001.tif]
